# SARS-CoV-2 infection prevalence on repatriation flights from Wuhan City, China

**DOI:** 10.1093/jtm/taaa135

**Published:** 2020-08-24

**Authors:** Hayley A Thompson, Natsuko Imai, Amy Dighe, Kylie E C Ainslie, Marc Baguelin, Sangeeta Bhatia, Samir Bhatt, Adhiratha Boonyasiri, Olivia Boyd, Nicholas F Brazeau, Lorenzo Cattarino, Laura V Cooper, Helen Coupland, Zulma Cucunuba, Gina Cuomo-Dannenburg, Bimandra Djaafara, Ilaria Dorigatti, Sabine van Elsland, Richard FitzJohn, Han Fu, Katy A M Gaythorpe, Will Green, Timothy Hallett, Arran Hamlet, David Haw, Sarah Hayes, Wes Hinsley, Benjamin Jeffrey, Edward Knock, Daniel J Laydon, John Lees, Tara D Mangal, Thomas Mellan, Swapnil Mishra, Andria Mousa, Gemma Nedjati-Gilani, Pierre Nouvellet, Lucy Okell, Kris V Parag, Manon Ragonnet-Cronin, Steven Riley, H Juliette T Unwin, Robert Verity, Michaela Vollmer, Erik Volz, Patrick G T Walker, Caroline Walters, Haowei Wang, Yuanrong Wang, Oliver J Watson, Charles Whittaker, Lilith K Whittles, Peter Winskill, Xiaoyue Xi, Christl A Donnelly, Neil M Ferguson

**Affiliations:** MRC Centre for Global Infectious Disease Analysis, Imperial College London, London, UK; MRC Centre for Global Infectious Disease Analysis, Imperial College London, London, UK; MRC Centre for Global Infectious Disease Analysis, Imperial College London, London, UK; MRC Centre for Global Infectious Disease Analysis, Imperial College London, London, UK; MRC Centre for Global Infectious Disease Analysis, Imperial College London, London, UK; MRC Centre for Global Infectious Disease Analysis, Imperial College London, London, UK; MRC Centre for Global Infectious Disease Analysis, Imperial College London, London, UK; NIHR Health Protection Research Unit in Healthcare Associated Infections and Antimicrobial Resistance, Imperial College London, London, UK; MRC Centre for Global Infectious Disease Analysis, Imperial College London, London, UK; MRC Centre for Global Infectious Disease Analysis, Imperial College London, London, UK; MRC Centre for Global Infectious Disease Analysis, Imperial College London, London, UK; MRC Centre for Global Infectious Disease Analysis, Imperial College London, London, UK; MRC Centre for Global Infectious Disease Analysis, Imperial College London, London, UK; MRC Centre for Global Infectious Disease Analysis, Imperial College London, London, UK; MRC Centre for Global Infectious Disease Analysis, Imperial College London, London, UK; MRC Centre for Global Infectious Disease Analysis, Imperial College London, London, UK; MRC Centre for Global Infectious Disease Analysis, Imperial College London, London, UK; MRC Centre for Global Infectious Disease Analysis, Imperial College London, London, UK; MRC Centre for Global Infectious Disease Analysis, Imperial College London, London, UK; MRC Centre for Global Infectious Disease Analysis, Imperial College London, London, UK; MRC Centre for Global Infectious Disease Analysis, Imperial College London, London, UK; MRC Centre for Global Infectious Disease Analysis, Imperial College London, London, UK; MRC Centre for Global Infectious Disease Analysis, Imperial College London, London, UK; MRC Centre for Global Infectious Disease Analysis, Imperial College London, London, UK; MRC Centre for Global Infectious Disease Analysis, Imperial College London, London, UK; MRC Centre for Global Infectious Disease Analysis, Imperial College London, London, UK; MRC Centre for Global Infectious Disease Analysis, Imperial College London, London, UK; MRC Centre for Global Infectious Disease Analysis, Imperial College London, London, UK; MRC Centre for Global Infectious Disease Analysis, Imperial College London, London, UK; MRC Centre for Global Infectious Disease Analysis, Imperial College London, London, UK; MRC Centre for Global Infectious Disease Analysis, Imperial College London, London, UK; MRC Centre for Global Infectious Disease Analysis, Imperial College London, London, UK; MRC Centre for Global Infectious Disease Analysis, Imperial College London, London, UK; MRC Centre for Global Infectious Disease Analysis, Imperial College London, London, UK; MRC Centre for Global Infectious Disease Analysis, Imperial College London, London, UK; MRC Centre for Global Infectious Disease Analysis, Imperial College London, London, UK; MRC Centre for Global Infectious Disease Analysis, Imperial College London, London, UK; School of Life Sciences, University of Sussex, Sussex, UK; MRC Centre for Global Infectious Disease Analysis, Imperial College London, London, UK; MRC Centre for Global Infectious Disease Analysis, Imperial College London, London, UK; MRC Centre for Global Infectious Disease Analysis, Imperial College London, London, UK; MRC Centre for Global Infectious Disease Analysis, Imperial College London, London, UK; MRC Centre for Global Infectious Disease Analysis, Imperial College London, London, UK; MRC Centre for Global Infectious Disease Analysis, Imperial College London, London, UK; MRC Centre for Global Infectious Disease Analysis, Imperial College London, London, UK; MRC Centre for Global Infectious Disease Analysis, Imperial College London, London, UK; MRC Centre for Global Infectious Disease Analysis, Imperial College London, London, UK; MRC Centre for Global Infectious Disease Analysis, Imperial College London, London, UK; MRC Centre for Global Infectious Disease Analysis, Imperial College London, London, UK; MRC Centre for Global Infectious Disease Analysis, Imperial College London, London, UK; MRC Centre for Global Infectious Disease Analysis, Imperial College London, London, UK; MRC Centre for Global Infectious Disease Analysis, Imperial College London, London, UK; MRC Centre for Global Infectious Disease Analysis, Imperial College London, London, UK; MRC Centre for Global Infectious Disease Analysis, Imperial College London, London, UK; Department of Mathematics, Imperial College London, London, UK; MRC Centre for Global Infectious Disease Analysis, Imperial College London, London, UK; Department of Statistics, University of Oxford, Oxford, UK; MRC Centre for Global Infectious Disease Analysis, Imperial College London, London, UK

The World Health Organization declared COVID-19 a global pandemic on 11 March 2020.^1^ Cases of atypical pneumonia caused by the SARS-CoV-2 virus were first detected in Wuhan City, China in late 2019. The growing scale of the outbreak and the strict travel and movement restrictions implemented in January 2020 prompted foreign governments to repatriate citizens from the then epicentre of transmission.^2^ Between 29 January and 27 February, 56 flights repatriated a total of 8597 individuals from Wuhan to 55 countries. This letter details SARS-CoV-2 infection prevalence over these repatriation flights. Estimating infection prevalence in repatriated individuals is useful, especially early in an outbreak of a novel pathogen when local case ascertainment at the origin is low and relies on symptomatic testing. For example, if infection prevalence in repatriates is high, this could indicate a highly transmissible and widely circulating pathogen.

**Figure 1:**
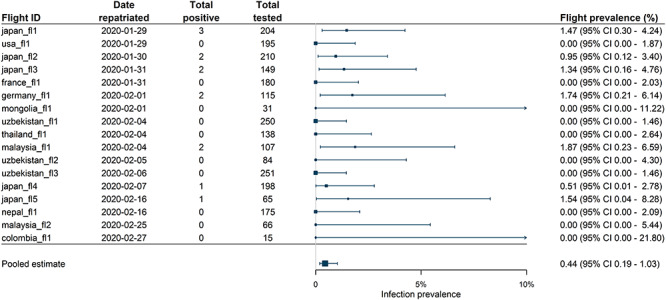
Observed per flight infection prevalence in repatriates returning from Wuhan China between 29 January to 27 February 2020 on flights where all individuals were tested on arrival and a pooled prevalence estimate across these flights. Pooled estimate results from binomial mixed-effect model fitting. Flights are ordered by date of departure. An arrow indicates where CIs for individual flight prevalence extends past 10%.

Repatriation flights were identified from international and local news outlets and government press releases. We tracked the total number of repatriates per flight, final destinations, number tested on arrival, during and before release from quarantine and of those who tested positive, the number symptomatic or asymptomatic where available (for downloadable data table of identified flights see our public GitHub repository: https://github.com/mrc-ide/repatriation-covid-19).

As testing protocols differed by country, we present the infection prevalence only for the 17 repatriation flights where all individuals (*N* = 2433) were tested upon arrival regardless of symptoms. As transmission during the flight itself could not be ruled out, we did not consider individuals who later tested positive during the quarantine period. By focusing on flights where all passengers were tested for SARS-CoV-2 infection with real-time reverse transcription polymerase chain reaction (RT-PCR), regardless of symptoms, a more accurate estimate of infection prevalence can be obtained compared with relying on symptomatic surveillance testing alone. We calculated the infection point prevalence per flight as the number of positive RT-PCR test results on arrival divided by the total population tested and the corresponding exact 95% binomial confidence intervals (CIs). We used a binomial mixed-effects model to obtain a pooled estimate of infection prevalence over this time frame, accounting for the heterogeneity between different repatriated populations.^3^^,^^4^

Per flight infection prevalence ranged from 0 to 1.9% and of the 2433 passengers tested immediately upon arrival, 13 individuals tested positive, resulting in a pooled infection prevalence in repatriates of 0.44% (95% CI: 0.19–1.03%) (Figure 1). Over the five flights leaving Wuhan between 30 January and 1 February inclusive (flights closest to the reported peak of the epidemic in Wuhan) where everyone was tested on arrival, the pooled infection prevalence was 0.88% (6/685, 95% CI: 0.39–1.93%). The infection risk for foreign nationals and tourists could differ from the general population due to socio-economic status, living and/or working conditions and exposure patterns. In addition, following the travel ban on 23 January symptomatic individuals may have been prevented from boarding these flights. Therefore, prevalence from repatriated flights can be considered a conservative estimate of infection prevalence in the wider population. Compared with the estimated infection prevalence of 3.6% (95% CI: 2.0–6.1%) and 6.3% (95% CI: 0.8–20.8%) amongst repatriates from European countries to Greece in late March, our estimates of prevalence in repatriates from Wuhan suggest relatively low levels of community transmission in Wuhan during this period despite flights occurring close to the reported peak of the epidemic.^5^

More accurately than PCR positivity in repatriated populations or symptomatic surveillance in local communities, retrospective local serological surveys can provide an insight into the scale of an outbreak as seroprevalence can be used as a measure of the cumulative incidence of infection. Several serological surveys have been conducted since the epidemic subsided in Wuhan. A survey conducted between 15 March and 28 April measured a seroprevalence of 3.27% (95% CI: 3.02–3.52%) in asymptomatic individuals visiting a general hospital in the Jianghan District, Wuhan, when adjusted for age and sex, and 2.72% (95% CI: 2.49–2.95%) when adjusted for assay sensitivity and specificity.^6^ Another survey conducted between 30 March and 10 April measured seroprevalence to be 3.8% (95% CI: 2.6–5.4%) in healthcare workers (HCWs), 3.8% (95% CI 2.2–6.3%) in hotel staff and 3.2% (95% CI: 1.6–6.4%) in family members of HCWs.^7^ However, it should be noted that these are high-risk populations and not necessarily representative of the general population of Wuhan. In addition, there is evidence that antibodies to SARS-CoV-2 wane quickly and so serosurveys may not capture all past infections within a population.^8^

The repatriation flights we considered represent a globally diverse population of foreign nationals who were residing in Wuhan City leading up to the outbreak for variable periods of time and for a variety of reasons: students, work-related travel, visiting friends and families and tourism. It is important to note that it is unclear how the risk of infection posed to these individuals compared with the risk of infection within the general population in Wuhan City. We assume the infection prevalence in repatriated individuals can be used as a lower bound for infection prevalence in the general population. While this assumption is hard to quantify and validate, it does impact the interpretation of our results and should be borne in mind. Despite this, characterizing infection prevalence from repatriated cohorts highlights a way to help bridge the gap between symptom-based surveillance that may underestimate true infection prevalence and seroprevalence surveys that are difficult to conduct during epidemic peaks.

## Author contributions

H.A.T., N.I., C.A.D., N.M.F. conceived the study; H.A.T., N.I., A.D., W.G., G.C.D., K.A.M.G., H.F. collected and extracted the international flight data and information on testing strategies; H.A.T. and N.I. carried out the analysis; H.A.T. wrote the first draft with input from N.I. and A.D.; all authors contributed to the final draft.

## Funding

Joint Centre funding from the UK Medical Research Council and Department for International Development (Grant number: MR/R015600/1).

**Conflict of interest**: All authors declare no competing interests.
